# Results of Endometrial Biopsy and Its Impact on Survival Data in Patients with High-Risk Uterine Sarcoma

**DOI:** 10.3390/jcm13144048

**Published:** 2024-07-11

**Authors:** Zaher Alwafai, Verena M. C. Reichert, Paula Spring, Marek Zygmunt, Günter Köhler

**Affiliations:** Department of Obstetrics and Gynecology, University Medicine Greifswald, Ferdinand-Sauerbruch-Straße, 17475 Greifswald, Germany

**Keywords:** uterine sarcoma, endometrial curettage, endometrial biopsy, histological findings, survival

## Abstract

**Background**: There are conflicting data regarding the detection rate of high-risk uterine sarcoma (HRUS) by endometrial biopsy. In addition, there are no studies in the literature on its impact on the chosen surgical approach and survival. **Methods**: This study includes 415 patients with HRUS. Of these, 178 (42.9%) patients had undergone endometrial biopsy. We analyzed the detection rate of endometrial biopsy and its impact on surgical approach and survival data. **Results**: Correct specific histologic diagnosis was achieved in only 30.0% of LMS and 33.3% of HGESS/UUS. Other uterine sarcoma, unspecified malignant mesenchymal tumor, carcinosarcoma or carcinoma were found in 45% of LMS and 78.2% of HGESS/UUS. As a result of the histologic findings, the rate of inadequate surgery was reduced by up to 19.9%. As tumor morcellation was performed significantly less often with biopsy (32.5% with vs. 55.4% without biopsy, *p* < 0.001), the locoregional recurrence-free interval remained unaffected between the two groups *(p* = 0.81). This is obviously an advantage of biopsy, although it does not affect the local recurrence rate in morcellated patients. **Conclusions**: Indicated endometrial biopsy is an important step in the diagnosis of HRUS, despite its low detection rate. It helps to avoid inappropriate surgical procedures but does not affect OS.

## 1. Introduction

High-risk uterine sarcomas (HRUS) are generally aggressive tumors with an unfavorable outcome [1]. The preoperative diagnosis of uterine sarcoma is difficult but important, especially to determine the choice of surgical approach. Patients with uterine leiomyosarcoma (LMS), high-grade endometrial stromal sarcoma (HGESS), and undifferentiated uterine sarcoma (UUS) have similar symptoms, including abnormal uterine bleeding (AUB), rapid uterine growth, and lower abdominal discomfort or pain [2,3,4]. Despite efforts, there is still no reliable diagnostic method to differentiate between uterine leiomyoma and sarcoma [5]. Numerous previous studies have described the challenges associated with sonographic diagnosis of uterine sarcoma [6,7]. In one of the largest multicenter studies regarding sonographic findings in uterine sarcoma involving 195 patients, uterine sarcomas were observed sonographically as solid masses with heterogeneous echogenicity, occasionally featuring irregular cystic areas, and were predominantly moderately or very well vascularized [8]. However, Köhler et al. [9] recently published a preoperative risk score that can significantly facilitate the diagnosis of uterine sarcoma based on medical history, clinical, and sonographic findings, and made recommendations for further diagnostic procedures.

Uterine LMS arise from the uterine muscle and are usually surrounded by the myometrium [9]. Therefore, performing endometrial biopsy in patients with LMS is considered less sensitive than in patients with other uterine malignancies such as endometrial carcinoma, carcinosarcoma, and adenosarcoma. However, HGESS and USS regularly develop in the endometrium and are therefore more amenable to endometrial diagnostic procedures. Curettage, endometrial sampling, and endometrial biopsy remain the most used diagnostic procedures in patients with abnormal uterine bleeding. These are different endometrial diagnostic procedures but are often used synonymously in the literature. Additional hysteroscopy improves sensitivity and should be performed when possible [2].

Even after endometrial biopsy, many uterine sarcomas are inadequately treated, mostly when histologically misdiagnosed as leiomyomas or other benign lesions. The evidence for the predictive value of endometrial biopsy in patients with endometrial cancer is well defined, while that for uterine sarcoma is still poorly defined [2,5]. However, the sensitivity of endometrial biopsy for uterine sarcoma, regardless of its indication, is unknown. In a meta-analysis of the results of endometrial biopsy for postmenopausal bleeding, one of the main symptoms of HRUS, uterine sarcoma is not mentioned at all [10].

As the efficacy of radiotherapy and chemotherapy is generally poor in uterine sarcoma, surgical therapy is considered the gold standard. The choice of surgical approach, whether minimally invasive or open with or without tissue morcellation, is a crucial point in the treatment, with controversial results regarding its influence on outcomes [2,5]. Raspagliesi et al. have investigated the impact of morcellation on survival outcomes in 125 patients with uterine sarcoma and reported that patients undergoing morcellation experienced a 3-fold increased risk of death [11]. Reichert et al. recently published a retrospective analysis of 301 LMS and showed that morcellation doubles the risk of locoregional recurrence (HR = 2.11, 95%-CI 1.41–3.16, *p* < 0.001) and surprisingly prolongs the time to distant metastases (HR = 0.52, 95%-CI 0.32–0.84, *p* = 0.008), while no influence on overall survival was observed [4]. Therefore, adequate surgery in terms of total hysterectomy without tumor injury remains the treatment of choice, while inadequate surgery must be strongly avoided [9].

Due to the very high number of endometrial biopsies performed for uterine bleeding [12] and the extremely low incidence of HRUSs of approximately 0.4 per 100,000 women, its sensitivity cannot be determined. Therefore, the primary outcome of this study is to determine the detection rate of endometrial biopsy in patients with HRUSs and its most common indications. Secondary outcomes include evaluating the impact of preoperative biopsy on surgical approach and the rate of inadequate surgery, as well as survival data.

## 2. Material and Methods

Materials: For this study, data on 467 patients with histologically proven high-risk uterine sarcomas (HRUS) by hysterectomy were retrospectively retrieved from the consultation database of the German Clinical Center of Excellence for Genital Sarcomas and Mixed Tumors (DKSM, University Medicine Greifswald, Germany). Inclusion criteria were patients with early or locally advanced stage leiomyosarcoma (LMS), high-grade endometrial stromal sarcoma (HGESS), or undifferentiated uterine sarcoma (UUS), who underwent hysterectomy between 2011 and 2021 with or without preoperative endometrial biopsy. Exclusion criteria were patients with metastatic uterine sarcoma, due to the influence of distant metastases on surgical procedure, postoperative management, and prognosis. Heterologous sarcomas and malignant mixed tumors were also excluded. Diagnoses and treatment were performed in different German in- and outpatient hospitals or clinics. Histologic specimens were also reviewed by various German pathology institutes.

Methods: Using available medical records, patient’s characteristics, symptoms, diagnostic procedures including sonographic findings, indications for endometrial biopsy, histopathological findings, following surgical approach, and survival data were collected and analyzed. Preoperative endometrial curettage was performed with or without hysteroscopy. Hysteroscopic findings were not analyzed and the role of hysteroscopy in the diagnosis was not evaluated in this study. A pipelle biopsy was not performed in any case. We used the term endometrial biopsy in the following. All histological findings of endometrial biopsy were categorized into clinically relevant benign and malignant groups, which could have an impact on the operative planning and were compared with the definitive final diagnosis of sarcoma.

Surgical interventions with high-power morcellation, tumor perforation (spontaneous or iatrogenic), or other tumor injury were considered as inadequate surgical procedures. In this study, the term AUB refers exclusively to intermenstrual bleeding and postmenopausal bleeding. Heavy uterine bleeding was not added to AUB here.

Because of the potential influence on prognosis and survival, data of menopausal status, tumor diameter, morcellation, adjuvant therapy including chemotherapy, and/or radiotherapy were determined in the two groups with and without endometrial biopsy.

Using SPSS version 27, OS rates of LMS and HGESS/UUS were determined by Kaplan–Meier analysis concomitant with the log-rank test. Differences in categorial variables were compared using the chi-square test or Fisher’s exact test and in continuous variables by means of the *t*-test. A *p*-value of <0.05 was considered statistically significant.

Missing clinical data were requested by the attending facility. All patients included gave their written consent to data collection and the use of anonymized personal records for research purposes. All procedures performed in this study were in accordance with the ethical standards of the local Institutional Research Committee of University Medicine (identifier: B 034/19) and with the 1964 Helsinki declaration and its later amendments.

## 3. Results

From all 467 patients, a total of 52 (11.1%) patients showed distant metastasis (58.1% with and 41.9% without endometrial biopsy) after further clinical investigations by preoperative or mainly postoperative imaging (CT, MRI). Of the remaining 415 HRUS, 303 (73.0%) were LMS and 112 (27.0%) HGESS/UUS. Because of the highly comparable clinical characteristics as well as prognosis and outcome, HGESS and UUS were combined into one group.

All patients underwent preoperative vaginal and partially abdominal sonography. An endometrial biopsy was performed in 42.9% (n = 178) of patients, 33% (n = 100) of LMS, and 69.6% (n = 78) of HGESS/UUS (Table 1). The frequency of AUB was significantly higher in HGESS/UUS (67.0%) than in LMS (49.8%) (*p* < 0.01). Solitary AUB was the main indication for endometrial biopsy in 48% of LMS and 50% of HGESS/UUS. A combination of bleeding events with other symptoms, abnormal intracavitary uterine sonographic findings, macroscopically visible tumors in the cervical canal such as polyps, or myoma in the statu nascendi were the indications in 41.0% of LMS and 35.9% of HGESS/UUS cases. Indications due to symptoms other than bleeding events, including heavy menstrual bleeding, were recorded in 11.0% of LMS and 14.1% of HGESS/UUS.

Sonographic uterine findings suspicious for sarcoma were described by Köhler et al. [9]. Only intracavitary sonographic suspicious findings were considered indications for biopsy. Intramural suspicious findings without AUB were not an indication for biopsy. Imaging procedures mostly after endometrial biopsy including the cohort of M1 were performed by CT (abdomen and/or chest) in 40.8%, MRI (abdomen) in 8.2%, CT plus MRI in 7.8%, chest-X-ray in 2%, and PET-CT in 1.2%. In only five cases, the indications of endometrial biopsy were the results of imaging procedures together with a suspicious sonographic finding.

A summary of the patients’ and tumors’ characteristics is shown in Table 1. The mean age (years) of women who underwent endometrial biopsy (56.3 in LMS and 58.9 in HGESS/UUS) was higher than that of women who did not undergo endometrial biopsy (52.1 in LMS and 55.7 in HGESS/UUS), reaching statistical significance only in the LMS group (*p* < 0.001). Postmenopausal status was observed in 49.3% (n = 100) of patients with LMS who did not receive endometrial biopsy and 66% (n = 66) of patients who did and in 64.7% (n = 22) of patients with HGESS/UUS without biopsy and 71.8% (n = 56) with biopsy. In summary, 51.5% (122) of patients with no biopsy and 68.5% (122) of patients with biopsy were postmenopausal.

The tumor diameter of LMS and HGESS/UUS was significantly lower in the biopsy group (8.86 and 8.68 cm) compared to the non-biopsy group (11.47 and 12.69 cm) (Table 1). Patients who underwent preoperative endometrial biopsy were significantly less likely to undergo inadequate surgery (28.0% (n = 28) of LMS and 38.5% (n = 30) of HGESS/UUS) than those who underwent surgery without biopsy (42.4% (n = 86) of LMS and 64.7% (n = 22) of HGESS/UUS). Morcellation of the tumor, the most common type of inadequate surgery, was significantly less common with biopsy at 21% (n = 21) of LMS and 11.5% (n = 9) of HGESS/UUS (Table 1) than without biopsy at 33% (n = 67) of LMS and 32.4% (n = 11) of HGESS/UUS; in summary, 16.9% (n = 30) with biopsy versus 32.9% (n = 78) without (chi-square test *p* < 0.001).

The specific preoperative histological diagnosis by endometrial biopsy was correctly made in 30.0% of LMS and in 33.3% of HGESS/UUS (Table 2).

The abovementioned histologic findings were categorized into clinically relevant groups (Table 3). Thus, the diagnosis of uterine sarcoma or unspecified malignant mesenchymal tumor was correct in 45.0% of LMS and 73.1% of HGESS/UUS. If misdiagnosed carcinosarcomas and unspecified carcinomas by endometrial biopsy were considered and added, the number of positive malignant findings increased to 78.2% of HGESS/UUS (Table 3).

Excluding the diagnosed variants of LM or STUMP, benign lesions (e.g., endometrial polyp adenomyosis, glandular hyperplasia, or endometrial stromal nodules) were diagnosed in 30.0% of LMS and 14.1% of HGESS/UUS. Normal histologic findings were described in 22.0% of LMS and only 7.7% of HGESS/UUS. Benign lesions and normal findings were significantly less frequent in HGESS/UUS (21.8%) than in LMS (52%) (*p* < 0.01).

**Figure 1:**
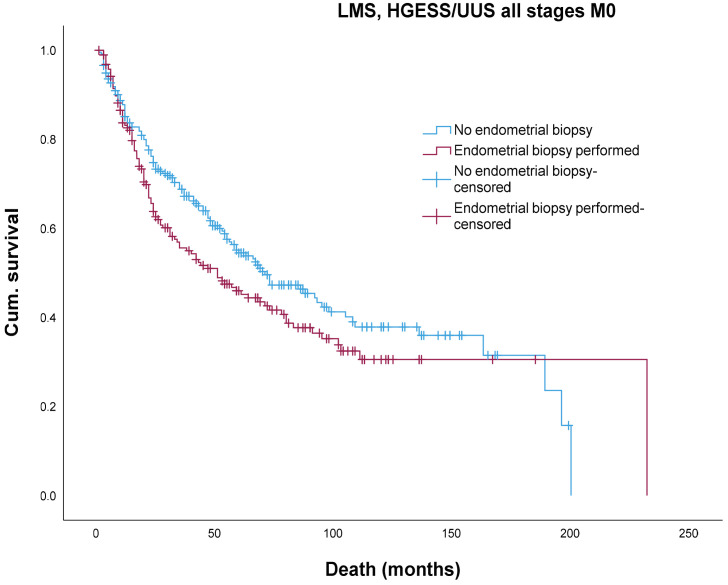
Impact of endometrial biopsy on OS in LMS and HGESS/USS.

There was no significant difference in the 5-year and 10-year overall survival rates in patients suffering from high-risk uterine sarcomas (LMS and HGESS/UUS) with (46.9% and 30.9%, respectively) or without (53.8% and 36.8%, respectively) preoperative endometrial biopsy (log rank 0.09) (Figure 1).

**Figure 2:**
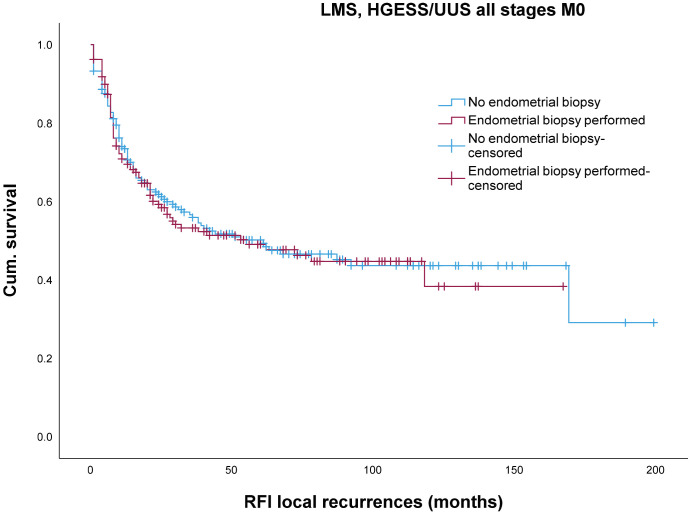
Impact of endometrial biopsy on locoregional relapse-free interval in LMS and HGESS/USS.

There was also no significant difference in the 5-year and 10-year locoregional recurrence-free interval (log rank = 0.81) (Figure 2).

**Figure 3:**
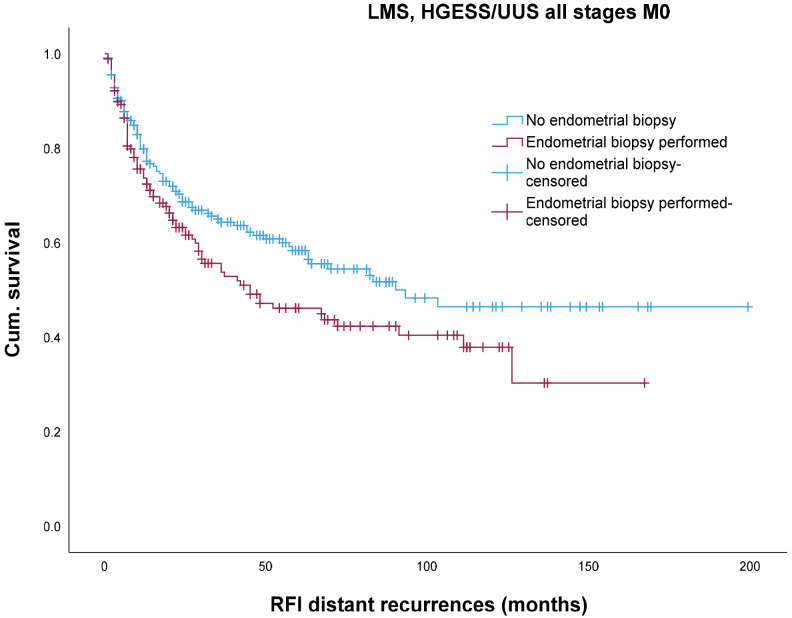
Impact of endometrial biopsy on distant relapse-free interval in LMS and HGESS/USS.

In contrast, the 5-year and 10-year distant recurrence-free interval was significantly higher in the non-biopsy group (58.3% and 46.4%, respectively) compared with the biopsy group (46.1% and 37.9%, respectively) (log rank = 0.046) (Figure 3), with a higher rate of distant metastases. In multivariable analysis, the distant metastasis-free interval was only dependent on intraoperative morcellation (HR 0.68, 95%-CI 0.48–0.96, *p* = 0.031) and postmenopausal status (HR 1.53, 95%-CI 1.11–2.1, *p* = 0.08), but not on the performed preoperative biopsy (HR 1.25, 95%-CI 0.92–1.69, *p* = 0.14).

In addition, we separately analyzed the impact of endometrial biopsy on survival data for LMS and HGESS/UUS (plots not shown). Regarding OS and locoregional recurrence-free interval, the data were consistent with those of all HRUS. An effect on 5- and 10-year distant metastasis-free interval was observed (56.4% and 37.6% without and 39.5% and 19.8% with biopsy, respectively) but was not statistically significant (log rank = 0.19).

Biopsy per se does not affect the local recurrence-free survival in morcellated and non-morcellated women (log rank *p* = 0.84). This finding is supported by the fact that in morcellated patients, biopsy also has no effect on local recurrence-free survival (log rank 0.361). In the non-biopsy group, morcellation is associated with significantly worse local recurrence-free survival as shown in univariate analysis (log rank < 0.001). However, in multivariate analysis, only morcellation (HR 1.76, CI 1.23–2.52, *p* = 0.002) and postmenopause (HR 1.48, CI 1.03–2.12, *p* = 0.03) had a significant influence on local recurrence-free survival, but not biopsy (HR 1.11, CI 0.77–1.58, *p* = 0.566).

A total of 28 patients received chemotherapy, 4 in the biopsy group and 24 in the non-biopsy group. In addition, 12 patients in each group received radiotherapy (brachytherapy with or without external beam radiation). The rate of chemotherapy and radiotherapy was too low to have an impact on the survival data.

## 4. Discussion

The detection rate of high-risk uterine sarcomas (HRUSs) by preoperative endometrial biopsy is relatively low. The correct specific histologic diagnosis could be achieved in only 30.0% of leiomyosarcoma (LMS) and 33.3% of high-grade endometrial stromal sarcoma and undifferentiated uterine sarcoma (HGESS/UUS). In fact, there are no prospective or large retrospective studies on the impact of endometrial biopsy in uterine sarcoma in general and in HRUSs in particular, regardless of the indication. There are limited retrospective data with relatively small numbers of cases on the detection rate of uterine sarcomas by endometrial biopsy. Most publications only refer to leiomyosarcoma.

A total of 178 women (42.9% of all patients) underwent endometrial biopsy prior to surgery. The numbers were significantly higher in patients with HGESS and UUS (69.6%) than in LMS (33.0%), confirming the fact that AUB occurs significantly more often when HGESS and UUS are diagnosed.

AUB as the main indication for endometrial biopsy is considered the main symptom in patients with uterine sarcoma. It indicates penetration of the sarcoma into the uterine cavity or primary development within the endometrium [13,14]. AUB has been described in 46% to 57% of LMS [15,16,17] and even more frequently in 56.4% to 65% of HGESS/UUS [15,18]. AUB was the main indication for endometrial biopsy in 48% of LMS and 50% of HGESS/UUS, while a combination of AUB and abnormal intracavitary sonographic findings or a macroscopically visible tumor in the cervical canal was the indication in 41.0% of LMS and 35.9% of HGESS/UUS. Other symptoms and findings, as well as heavy menstrual bleeding, were the indication for endometrial biopsy in 11.0% of LMS and 14.1% of HGESS/UUS.

According to our data, AUB occurred mostly as intermenstrual or postmenopausal bleeding in about half of the patients with LMS (49.8%) and significantly more frequently in patients with HGESS/UUS (67.0%) (*p* < 0.01). This difference is because HGESS and UUS usually arise in the endometrium and spread very quickly into the uterine cavity. In contrast, the majority of LMS are located primarily in the myometrium and invade the cavity at a later stage [13,14].

Our analysis showed that the correct specific histologic diagnosis by endometrial biopsy was unsatisfactorily low in 30.0% and 33.3% of LMS and HGESS/UUS, respectively. From the pathologists’ point of view, a correct diagnosis based on endometrial biopsy could be achieved in only 30% of cases [19]. However, if the results refer to all malignant uterine tumors (including carcinosarcomas and carcinomas) detected by endometrial biopsy, the detection rate is significantly higher with up to 45.0% in LMS and 78.2% in HGESS/UUS. Relevant preoperative misdiagnosis (benign lesions and normal findings) was observed in 52.0% of LMS and 21.8% of HGESS/UUS.

Kho et al. recently published a similar analysis of 79 patients with uterine LMS who underwent endometrial sampling with or without diagnostic hysteroscopy followed by hysterectomy; 31.6% of LMS could be diagnosed preoperatively by endometrial sampling alone and 66.7% when sampling was combined with hysteroscopy [2]. The impact of diagnostic hysteroscopy on the detection rate of endometrial biopsy was not evaluated in this study.

Hinchcliff et al. evaluated the role of endometrial biopsy in the preoperative detection of 68 uterine LMS and showed that 51.5% of cases were diagnosed preoperatively as LMS or spindle cell proliferation. Only 35% of cases were correctly diagnosed as uterine LMS [20]. In the study of Skorstad et al., 142 of 209 women (67.9%) with LMS underwent preoperative endometrial biopsy. A malignant finding without a specific diagnosis was described in 38.7% of cases [21]. In the study of Bansal et al., preoperative endometrial sampling suggested an invasive tumor in 86% and could predict the general histologic diagnosis of sarcoma in only 64%, but not the specific entity [14]. Therefore, an exact comparison with our data was not possible.

Studies and case reports of endometrial biopsy for stromal sarcoma are even rarer than for LMS. Jin et al. reported only 23 cases of UUS and 47 cases of LGESS; endometrial biopsy was performed in only 65% and 29.8% of cases, respectively. Overall, the correct diagnosis was achieved in 89.6% of this small group [22].

Li et al. described 114 cases of uterine sarcoma. Preoperative endometrial biopsy was performed in 48 patients. A diagnosis of uterine malignancy was made in 36 patients (75%) [23].

In a previous study of the French Sarcoma Group, data from 39 patients with HGESS and UUS were analyzed. Only 15 patients (38.5%) underwent preoperative endometrial biopsy, and 7 patients (46.6%) were found to have uterine sarcoma [18].

Tanner et al. studied 21 uterine sarcomas (HGESS and UUS); 11 patients (50%) received endometrial biopsy. The pathological diagnosis was correct in seven (64%) of the analyzed tumors [24].

Wais et al. identified 302 patients with various uterine sarcomas, and 114 (51%) underwent endometrial sampling. There was evidence of sarcoma in 65% of cases. The main indications for this procedure were AUB including PMB [25].

Despite efforts, there are still no reliable diagnostic procedures to differentiate between uterine leiomyoma and sarcoma [5]. Transcervical core needle biopsycan only be applied in order to achieve clarification in cases in which the diagnosis remains uncertain despite having performed all of the known diagnostic measures [26,27]. However, this is definitely not a diagnostic method for intermenstrual and postmenopausal bleeding. Nevertheless, experiences with this approach have been surprisingly positive in selected cases. In one study, transcervical core needle biopsies were taken from 453 women whose tumors were LMS-suspicious (high SI in T1/2 W MRI and/or high LDH-values and/or rapidly growing tumor with 3-fold gain in volume within one year) [26]. Among 372 eligible cases, only seven sarcomas were found. Nonetheless, false-negative results must be reconsidered, in particular for spindle-cell sarcomas [28]. One reason for this is because mitoses, atypia, and necroses can in fact be concentrated in so-called “hot spots”. There is a real risk of missing these hot spots using core biopsy, so that, essentially, only positive findings can be truly relied on.

According to our data, the preoperative histologic diagnosis of sarcoma has an important impact on the mode of sarcoma surgery, whereas misdiagnosis could lead to incorrect surgical treatment, resulting in suboptimal therapeutic approaches, prolonging the operative time, or in some cases leading to a second surgical procedure. As expected after endometrial biopsy, the indication of sarcoma for surgery increased from 26.4% to 57% in LMS and from 38.3% to 84.6% in HGESS/UUS. Consistently, the number of inadequately treated patients decreased from 42.4% to 28% in LMS and from 64.7% to 38.5% in HGESS/UUS (*p* < 0.05 for both groups). The number of morcellated sarcomas decreased significantly from 33.0% to 21% in LMS (*p* = 0.016) and from 32.4% to 11.5% in HGESS/UUS (*p* = 0.017). To the best of our knowledge, there are no studies in the literature on the impact of preoperative endometrial biopsy on surgical approach.

However, based on our data, preoperative endometrial biopsy does not affect overall survival in patients with HRUS. Even when the histologic result of endometrial biopsy was considered whether malignant or non-malignant, no significant difference in overall survival was seen in both groups. This is consistent with the results published by Kho et al. with a median overall survival of 50 months in LMS and no significant difference between patients diagnosed before or after hysterectomy [2]. The biopsy group in our study had more prognostically favorable variables, such as a smaller tumor diameter and lower morcellation rate, than unfavorable variables, such as postmenopausal status (the latter only for LMS) (Table 1).

The correlation between intrabdominal morcellation of uterine sarcoma and outcome were reported previously [5,11]. However, only a few studies have evaluated the long-term outcomes in these patients, with a clear impact on disease-free interval and very heterogenous results regarding overall survival [4,29,30]. Similar results regarding morcellation and outcomes in uterine smooth muscle tumor of uncertain malignant potential were demonstrated [31]. Morcellation intraabdominally should be avoided, if the diagnosis of sarcoma could not be excluded, otherwise specimen retrieval via a mini-laparotomic incision seems to be a suitable alternative [32].

The effect of endometrial biopsy on recurrence-free interval has not been investigated in the literature. As shown in a study from our group, tumor morcellation doubles the risk of locoregional recurrence rate [4]. As tumor morcellation was performed significantly less often with biopsy (32.5% with vs. 55.4% without biopsy, *p* < 0.001), the locoregional recurrence-free interval remained unaffected between the two groups (log rank = 0.81). Regarding morcellation, biopsy per se does not affect the local recurrence-free survival in morcellated and non-morcellated women (log rank *p* = 0.84). In the non-biopsy group, morcellation is associated with significantly worse local recurrence-free survival as shown in univariate analysis (log rank < 0.001) (graphs not shown). However, in multivariate analysis, only morcellation (HR 1.76, CI 1.23–2.52, *p* = 0.002) and postmenopause (HR 1.48, CI 1.03–2.12, *p* = 0.03) but not the biopsy significantly influenced the local recurrence-free survival (HR 1.11, CI 0.77–1.58, *p* = 0.566). The reduction in the morcellation rate is obviously an advantage of biopsy, although it does not influence the local recurrence rate of morcellated patients. As was also shown in a previous study [4], tumor morcellation significantly halves the risk of distant metastases (HR = 0.52). This may explain why the distant metastasis-free interval is longer without biopsy, because the proportion of morcellated tumors is significantly higher in this group. But, in the multivariable analysis, distant metastasis was only dependent on morcellation (HR 0.68, 95%-CI 0.48–0.96, *p* = 0.031) and postmenopausal status (HR 1.53, 95%-CI 1.11–2.1, *p* = 0.08) but not on biopsy performed (HR 1.25, 95%-CI 0.92–1.69, *p* = 0.14). As the proportion of postmenopausal women in both groups is not significantly different, only the effect of morcellation on the recurrence-free interval of distant metastases remains. The phenomenon of morcellation on distant metastases cannot be explained at present and has already been discussed in the work of Reichert et al. [4]. Similar results were reported by Nobre [33].

Due to the small number of cases who underwent radiotherapy or chemotherapy, and their unproven efficacy, chemotherapy and radiotherapy were not included or considered in this analysis.

Strengths and limitations of this study: Our study used centralized data and was limited by its observational retrospective nature, which may introduce selection bias. The data were collected from several centers in Germany and the histological examination was performed in different institutions, which limits our ability to identify factors that may affect the quality of the results. As mentioned in the Section 2, the comparison with the current literature was limited by the fact that our study only examined complete endometrial curettage, whereas other groups used different methods of analysis, such as endometrial sampling or endometrial biopsy, according to the literature.

## 5. Conclusions

The detection rate of histologically proven high-risk uterine sarcoma (HRUS) by endometrial biopsy is up to 33%; meanwhile, its sensitivity is unknown when endometrial biopsy was performed due to abnormal uterine bleeding or abnormal intracavitary sonographic finding. Uterine malignancies, including misdiagnosed carcinomas and carcinosarcomas, were detected in 45.0% of LMS and 78.2% of HGESS/UUS by endometrial biopsy.

The preoperative diagnosis of HRUSs by endometrial biopsy has a significant impact on the surgical procedure. Therefore, inappropriate surgery, mainly in the form of tumor morcellation, is performed significantly less. Preoperative endometrial biopsy does not affect OS. By reducing the rate of tumor morcellation, endometrial biopsy reduces the risk of the locoregional recurrences induced by this inadequate procedure. This appears to be a benefit of biopsy. According to the available data, the distant recurrence-free interval is only indirectly prolonged in the non-biopsy group.

Endometrial biopsy is a cornerstone in the management of AUB, no matter whether sarcoma or other malignant uterine tumors are expected. However, benign or normal findings, observed in up to 52% of LMS and 21.8% of HGESS/UUS, can also provide a false sense of security. To minimize the rate of false negative findings, only clearly indicated endometrial biopsies should be performed.

## Figures and Tables

**Table 1:** Patient’s and tumor’s characteristics with and without endometrial biopsy.

	No Endometrial Biopsy	Endometrial Biopsy	*p*-Value
**Leiomyosarcoma (LMS)** **all stages, n = 303**	**n = 203 (67.0%)**	**n = 100 (33.0%)**	
Age in years (mean)	52.1	56.3	0.001
Postmenopause	100 (49.3%)	66 (66.0%)	0.016
Tumor diameter (mean)	11.47	8.86	0.000
Morcellation	67 (33%)	21 (21%)	0.016
**High-grade endometrial stromal sarcoma/Undifferentiated uterine sarcoma (HGESS/UUS)** **all stages, n = 112**	**n = 34 (30.4%)**	**n = 78 (69.6%)**	
Age in years (mean)	55.7	58.9	0.190
Postmenopause	22 (64.7%)	56 (71.8%)	0.453
Tumor diameter (mean)	12.69	8.68	0.006
Morcellation	11 (32.4%)	9 (11.5%)	0.017

**Table 2:** Histological findings of preoperative endometrial biopsy.

			LMS	HGESS/UUS	
Histological findings	Leiomyoma (LM)	n	15	2	17
		%	15.0%	2.6%	
	Variants of LM, uterine smooth muscle tumors of uncertain malignant potential (STUMP)	n	3	0	3
		%	3.0%	0.0%	
	LGESS	n	1	1	2
		%	1.0%	1.3%	
	LMS	n	30	4	34
		%	30.0%	5.1%	
	Not specified high-grade-sarcoma	n	1	6	7
		%	1.0%	7.7%	
	HGESS	n	1	19	20
		%	1.0%	24.4%	
	UUS	n	0	7	7
		%	0.0%	9.0%	
	Adenosarcoma	n	0	2	2
		%	0.0%	2.6%	
	Carcinosarcoma	n	0	1	1
		%	0.0%	1.3%	
	Not specified sarcoma	n	9	6	15
		%	9.0%	7.7%	
	Not specified stromal sarcoma	n	0	7	7
		%	0.0%	9.0%	
	Unclear malignant tumor	n	3	5	8
		%	3.0%	6.4%	
	Unclear findings or necrosis	n	0	2	2
		%	0.0%	2.6%	
	Undifferentiated carcinoma	n	0	1	1
		%	0.0%	1.3%	
	Endometrial cancer	n	0	2	2
		%	0.0%	2.6%	
	Not specified benign tumor	n	8	6	14
		%	8.0%	7.7%	
	Normal findings	n	22	6	28
		%	22.0%	7.7%	
	Endometrial polyp	n	6	0	6
		%	6.0%	0.0%	
	Adenomyosis/adenomyoma	n	1	0	1
		%	1.0%	0.0%	
	Glandular hyperplasia	n	0	1	1
		%	0.0%	1.3%	
Total			100	78	178
		%	100.0%	100.0%	

**Table 3:** Reclassification into clinically relevant groups of histological findings of preoperative endometrial biopsy.

			LMS	HGESS/UUS	
Histological findings	Sarcoma or not specified malignant mesenchymal tumor	n	45	57	103
		%	45.0%	73.1%	
	Carcinosarcoma or not specified carcinoma	n	0	4	3
		%	0.0%	5.1%	
	Variants of LM, STUMP	n	3	0	3
		%	3.0%	0.0%	
	Benign tumor	n	30	11	41
		%	30.0%	14.1%	
	Normal findings	n	22	6	28
		%	22.0%	7.7%	
Total		n	100	78	178
		%	100.0%	100.0%	

## Data Availability

Data are contained within the article.
